# A new genotype of bovine leukemia virus in South America identified by NGS-based whole genome sequencing and molecular evolutionary genetic analysis

**DOI:** 10.1186/s12977-016-0239-z

**Published:** 2016-01-12

**Authors:** Meripet Polat, Shin-nosuke Takeshima, Kazuyoshi Hosomichi, Jiyun Kim, Taku Miyasaka, Kazunori Yamada, Mariluz Arainga, Tomoyuki Murakami, Yuki Matsumoto, Veronica de la Barra Diaz, Carlos Javier Panei, Ester Teresa González, Misao Kanemaki, Misao Onuma, Guillermo Giovambattista, Yoko Aida

**Affiliations:** Viral Infectious Diseases Unit, RIKEN, 2–1 Hirosawa, Wako, Saitama 351-0198 Japan; Laboratory of Viral Infectious Diseases, Department of Computational Biology and Medical Sciences, Graduate School of Frontier Science, The University of Tokyo, Wako, Saitama 351-0198 Japan; Department of Bioinformatics and Genomics, Graduate School of Medical Sciences, Kanazawa University, Takara-machi 13-1, Kanazawa, Ishikawa 920-8640 Japan; LAVET, Carampangue 595, Valdivia, Chile; Department of Virology, Faculty of Veterinary Sciences, National University of La Plata, 60 and 118, CC 296, 1900 La Plata, Argentina; Institute for Animal Science, Shitara-cho, Aichi 441-2433 Japan; IGEVET, CCT La Plata-CONICET, Facultad de Ciencias Veterinarias, National University of La Plata, 60 and 118, CC 296, 1900 La Plata, Argentina

**Keywords:** Bovine leukemia virus (BLV), Next generation sequencing, BLV whole genome analysis, gp51 *env* sequencing, Phylogenetic analysis, South America

## Abstract

**Background:**

Bovine leukemia virus (BLV) is a member of *retroviridae* family, together with human T cell leukemia virus types 1 and 2 (HTLV-1 and -2) belonging to the genes *deltaretrovirus*, and infects cattle worldwide. Previous studies have classified the *env* sequences of BLV provirus from different geographic locations into eight genetic groups. To investigate the genetic variability of BLV in South America, we performed phylogenetic analyses of whole genome and partial *env* gp51 sequences of BLV strains isolated from Peru, Paraguay and Bolivia, for which no the molecular characteristics of BLV have previously been published, and discovered a novel BLV genotype, genotype-9, in Bolivia.

**Results:**

In Peru and Paraguay, 42.3 % (139/328) and over 50 % (76/139) of samples, respectively, were BLV positive. In Bolivia, the BLV infection rate was up to 30 % (156/507) at the individual level. In Argentina, 325/420 samples were BLV positive, with a BLV prevalence of 77.4 % at the individual level and up to 90.9 % at herd level. By contrast, relatively few BLV positive samples were detected in Chile, with a maximum of 29.1 % BLV infection at the individual level. We performed phylogenetic analyses using two different approaches, maximum likelihood (ML) tree and Bayesian inference, using 35 distinct partial *env* gp51 sequences from BLV strains isolated from Peru, Paraguay, and Bolivia, and 74 known BLV strains, representing eight different BLV genotypes from various geographical locations worldwide. The results indicated that Peruvian and Paraguayan BLV strains were grouped into genotypes-1, -2, and -6, while those from Bolivia were clustered into genotypes-1, -2, and -6, and a new genotype, genotype-9. Interestingly, these results were confirmed using ML phylogenetic analysis of whole genome sequences obtained by next generation sequencing of 25 BLV strains, assigned to four different genotypes (genotypes-1, -2, -6, and -9) from Peru, Paraguay, and Bolivia. Comparative analyses of complete genome sequences clearly showed some specific substitutions, in both structural and non-structural BLV genes, distinguishing the novel genotype-9 from known genotypes.

**Conclusions:**

Our results demonstrate widespread BLV infection in South American cattle and the existence of a new BLV genotype-9 in Bolivia. We conclude that at least seven BLV genotypes (genotypes-1, -2, -4, -5, -6, -7, and -9) are circulating in South America.

**Electronic supplementary material:**

The online version of this article (doi:10.1186/s12977-016-0239-z) contains supplementary material, which is available to authorized users.

## Background

Bovine leukemia virus (BLV) is a member of *retroviridae* family belonging to the genes *deltaretrovirus*, being considered a model of pathogens for human T-cell leukemia virus types 1 (HTLV-1) [1], and is the etiological agent of enzootic bovine leukosis (EBL), the most common neoplastic disease of cattle [2, 3]. Some cattle infected with BLV suffer from lymphomas and/or B-lymphocyte proliferation (persistent lymphocytosis), but the majority of BLV-infected cattle are healthy carriers of the virus [2, 3].

The complete genome of BLV consists of 8714 nucleotides, including the structural and enzymatic *gag*, *pro*, *pol*, and *env* essential genes and two identical long terminal repeats (LTRs). The BLV *gag* gene is translated as the precursor, Pr70 Gag, and processed into three mature proteins: the matrix protein, p15 (MA), the most abundant capsid protein, p24 (CA), and the nucleocapsid protein, p12 (NC) [4, 5]. The BLV *pro* and *pol* genes encode proteases (Pro) p14 and p80, harboring reverse transcriptase (RT) and integrase (IN) activities, respectively [3, 4]. The *env* gene encodes a mature surface glycoprotein (gp51) and a transmembrane protein (gp30) [4], and is involved in viral infectivity [6–8]. In addition, the BLV genome contains a pX region, located between the *env* sequence and the 3′ LTR [2, 3]. At least four proteins, including the regulatory proteins Tax and Rex, and the accessory proteins R3 and G4, are encoded by this genomic region. The Tax protein has been extensively studied and is believed to play a critical role in BLV induced leukemogenesis [9]. Rex is responsible for nuclear export of viral RNA and promotes cytoplasmic accumulation and translation of viral messenger RNA (mRNA) in BLV-infected cells [10]. The R3 and G4 proteins contribute to the maintenance of high viral load [11, 12] and the G4 protein is particularly relevant to leukemogenesis, since it can immortalize primary embryonic fibroblasts [13]. The R3 protein contributes to the maintenance of infectivity [12], and is located in the nucleus and cellular membranes [13]. In addition to the above, BLV RNA polymerase III (pol III)-encoded viral microRNAs are strongly expressed in preleukemic and malignant cells, in which structural and regulatory gene expression is repressed, suggesting a possible key role in tumor onset and progression [14, 15].

The Env gp51 glycoprotein plays an essential role in the viral life cycle [7, 8]. gp51 is required for cell entry and the target of neutralizing antibodies [8, 16]. The N-terminal half of BLV gp51 contains three conformational epitopes, F, G and H [17], and plays an important role in viral infectivity and syncytium formation [7, 18, 19], while the C-terminal half of BLV gp51 contains the linear epitopes A, B, D, and E [16, 17]. Therefore, the gp51 region has been widely used for BLV genotyping studies and recent phylogenetic studies of this region from viral strains isolated worldwide demonstrate that BLV can be classified into at least eight genotypes [20–33].

Cattle were introduced to the American continent by the Spanish conquerors from 1493. Within a few years, these cattle had spread all over South America and their population size had increased to several million [34]. More than 300 years, Creole cattle were the only cattle bred in America, and during the end of 19th and the beginning of the 20th centuries, animals from British breeds (Shorthorn, Hereford, Angus, and Holstein) and indicus breed (Nelore, Brahman, and Gir) were introduced in temperate/cold and subtropical/tropical regions, respectively, in order to improve beef and dairy production through crossbreeding with local populations or directly by replacement. BLV infects cattle worldwide, imposing a severe economic impact on the dairy cattle industry, and EBL has been recognized in South America (Brazil) since 1943 [35]. World Organization for Animal Health data (OIE 1999 and 2009) has confirmed the presence of BLV in most parts of South America. In Brazil, infection rates between 17.1 and 60.8 % were detected, with individual prevalence rates varying considerably among states, and recent data indicate a sharp increase in the prevalence of BLV [20, 23, 35–38]. Early phylogenetic analysis of the BLV *env* gene demonstrated that Brazilian BLV isolates were clustered into genotypes-1, -2, -5, -6, and -7 [20, 23]. In Columbia, BLV prevalence levels up to 83.3 % have been reported, with average prevalence differing significantly between cattle breeds and according to the viral detection method used [39, 40]. Previous studies have indicated the presence of BLV in Argentina [26, 31, 41, 42], with individual and herd prevalence levels of 32.8 and 84 %, respectively [43]. The presence of BLV genotypes-1, -2, -4, and -6 were confirmed in Argentina [26, 31, 32]. BLV infection has also been reported in Peru, Chile, and Uruguay [24, 33, 44]. Restriction fragment length polymorphism (RFLP) analysis of Chilean BLV strains demonstrated the presence of genotypes-1 and -4 [24]; however, a study of cattle in Uruguay and Chile by Moratorio et al. [33], aligning partial *env* gp51 sequences with data available from other South American BLV strains, found that Chilean BLV strains were assigned into genotypes-4 and -7, but not -1, while Uruguayan BLV strains were of genotype-1. Thus, it appears that at least six genotypes of BLV strains are circulating in South America. However, there are currently no studies of the molecular characteristics of BLV in Bolivia, Paraguay, or Peru (OIE 2009).

Here, we investigated the distribution of and molecularly characterized BLV strains in Peru, Paraguay, Bolivia, Argentina, and Chile, together with other South American BLV strains as follows: (1) first, the spread of BLV infection was investigated by amplification of BLV LTRs by nested polymerase chain reaction (PCR) from blood samples obtained from a total of 2204 cattle in Peru, Paraguay, Bolivia, Argentina, and Chile; (2) second, the genetic variability of BLV strains circulating in Peru, Paraguay, and Bolivia was examined by DNA sequencing and 35 distinct strains collected from these countries were classified by genotype using phylogenetic analyses based on partial *env* (gp51) sequences, comparing these strains and isolates from other geographical locations worldwide; (3) third, whole genome sequences of 25 BLV strains, assigned to four different genotypes, from Peru, Paraguay and Bolivia were obtained by next generation sequencing (NGS) and compared with eight full-length BLV genome sequences, generated by Sanger sequencing, available in the NCBI database.

Collectively, our comprehensive studies provide strong evidence for the existence of a novel BLV genotype, which we designate genotype-9, in Bolivia.

## Results

### Investigation of the spread of BLV infection in South America

**Fig. 1:**
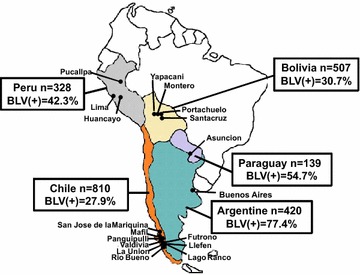
Map of South America showing the number of cattle (head) sampled from countries included in the study. The five countries and cities/municipalities where sampling was performed are indicated in the figure. *n* indicates the total number of samples in each country and *BLV*(*+*) % indicates the BLV prevalence rate

To investigate the spread of BLV infection in South America, a total of 2204 blood samples were collected from cattle in different regions of five South American countries, including Peru, Paraguay, Bolivia, Argentina, and Chile, as shown in Fig. 1. The samples were screened for BLV infection by nested PCR to amplify BLV LTRs (Table 1; Fig. 1). In Peru, samples from 139 cattle out of 328 (42.3 %) were positive for the BLV provirus, with prevalence rates ranging from 0 to 58.6 % at the farm level. In Paraguay, 76 cattle samples out of 139 (54.5 %) were BLV positive and infected cattle breeds were Holstein, Brown Swiss, or Holstein × Brown Swiss. All breeds showed a high prevalence of BLV infection in Paraguay, with a range of 28.6–71.4 % in the Holstein breed and 36.4 % in Brown Swiss. Of 507 samples collected from eight farms in different parts of Bolivia, 156 (30.7 %) were positive for BLV provirus. Infected cattle breeds in Bolivia were Holstein, Gir and Yacumeño, but not Brown Swiss, Brahman and Montana. Interestingly, BLV infection levels differed from farm to farm, and from breed to breed. For example, Holstein cattle showed the highest BLV prevalence, with up to 100 % at the individual level, while Yacumeño showed moderate levels of BLV infection ranging from 4.5 to 24.3 %. Compared to the above-mentioned countries, samples collected from Argentina (n = 420) demonstrated extremely high levels of BLV prevalence (77.4 %), with up to 90.9 % at the herd level. In Chile, 810 samples were collected from multiple cattle breeds at 19 farms over a relatively wide geographical area (Table 1). Of the samples screened, 236 (29.1 % prevalence) were BLV provirus positive. It is difficult to compare BLV prevalence levels between breeds in Chile, as a wide range of breeds were BLV positive; however, remarkable differences were observed between farms, breeds, and locations, indicating variability of BLV infection in this country. Our study result is in agreement with previous study [45, 46] that on average dairy cattle breeds, such as Holstein and Brown Swiss, has higher level of BLV infection than that of beef cattle, namely Yacumeño and Nelore. However, there is no statistically significant correlation of BLV prevalence among Breeds, because individually breeds are different from farm to farm and also from country to county.

**Table 1:** Summary of BLV detection by nested PCR for five South American countries

Country	City/municipality	Farm	Breed	Positive % (+/All)^a^
Peru	Lima	A	Holstein	58.6 (61/104)
	Pucallpa	B	Brahman × Nelore	40.0 (78/195)
	Huancayo	C	Holstein	0.0 (0/29)
Paraguay	Asuncion	A	Holstein	71.4 (25/35)
		B	Holstein	60.0 (18/30)
		C	Holstein	61.3 (19/31)
		D	Holstein	33.3 (1/3)
		E	Holstein	28.6 (8/28)
			Brown Swiss	36.4 (4/11)
			Holstein × Brown Swiss	100.0 (1/1)
Bolivia	Yapacani	A	Yacumeño	27.7 (10/36)
		B	Yacumeño	19.0 (4/21)
		C	Yacumeño	4.5 (1/22)
			Brown Swiss	0.0 (0/1)
		D	Yacumeño	24.3 (7/29)
	Montero	E	Holstein	38.0 (19/50)
		F	Gir	18.5 (20/108)
			Nelore	20.0 (21/105)
	Portachuelo	G	Holstein	64.2 (70/109)
	Santacruz	H	Brahman	0.0 (0/3)
			Gir	0.0 (0/2)
			Holstein	100.0 (1/1)
			Montana	0.0 (0/2)
			Nelore	9.0 (1/11)
			Brahman × Nelore	28.6 (2/7)
Argentina	Buenos Aires	D	Holstein	90.9 (60/66)
		E	Holstein	79.4 (152/194)
		F	Holstein	70.6 (113/160)
Chile	Valdivia	A	Overo Colorado	12.0 (3/25)
		B	Chilean Wagyu	50.0 (1/2)
			Simmental	0.0 (0/1)
			Jersey	0.0 (0/1)
			Overo Colorado	0.0 (0/5)
			Overo Negro	0.0 (0/2)
			Red Angus	0.0 (0/2)
		C	Overo Colorado	100.0 (30/30)
	Mafil	D	Holstein	0.0 (0/4)
			Overo Colorado	3.1 (1/32)
		E	Holstein	30.0 (9/30)
		F	Hereford × Overo Colorado	30.0 (6/20)
	San Jose de la Mariquina	G	Hereford × Jersey	91.7 (66/72)
		H	Holstein	50.0 (10/20)
	Panguipulli	I	Overo Colorado	7.7 (6/13)
			Overo Negro	30.0 (10/33)
		J	Overo Colorado	16.6 (1/6)
			Overo Negro	38.5 (20/52)
	Rio Bueno	K	Holstein	12.1 (4/33)
		L	Holstein	39.3 (11/28)
	Lago Ranco	M	Black Angus	0.0 (0/17)
			Red Angus	0.0 (0/7)
		N	Overo Colorado	10.5 (2/19)
	La Union	O	Overo Negro	5.0 (1/20)
	Futrono	P	Black Angus	41.7 (15/36)
			Red Angus	19.7 (12/61)
		Q	Chilean Wagyu	2.5 (2/79)
			Hereford	0.0 (0/1)
			Red Angus	12.5 (2/16)
		R	Hereford × Angus	14.7 (14/95)
	Llefen	S	Hereford	20.8 (10/48)

^a^(+/All) indicates the ratio of the number of BLV positive samples to that of all samples

### Identification of a novel BLV genotype, genotype-9, by phylogenetic analysis of partial *env* gp51 sequences

**Fig. 2:**
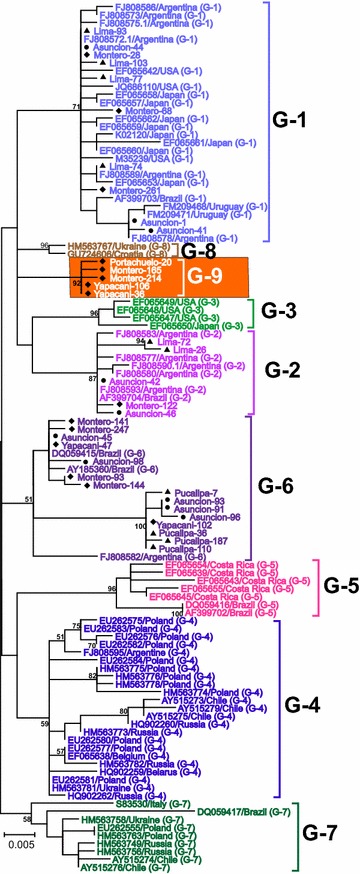
Maximum likelihood phylogenetic tree of BLV *env* partial sequences from different geographical locations worldwide. A maximum likelihood phylogenetic tree was constructed from 35 distinct 475 bp BLV *env* sequences (submitted to the GenBank nucleotide sequence database and assigned accession numbers LC075543–LC075577) and with 74 sequences from known BLV strains, representing eight different BLV genotypes from other South American countries, including Argentina, Brazil, Chile, and Uruguay, and other parts of the world. These 74 nucleotide sequences were obtained from the GenBank nucleotide sequence database. The South American BLV strains in this study are indicated by sample ID and location of collection. Other isolates are shown in the tree by accession number and country of origin. South American BLV strains in this study are marked according to the country of origin as follows: Peru (*filled triangle*), Paraguay (*filled circle*), and Bolivia (*filled diamond*). Genotypes are indicated by *numbers* to the *right* of the figure. Genotypes-1, -2, -3, -4, -5, -6, -7, -8 and -9 are shown in *pastel blue*, *violet*, *sea green*, *blue*, *pink*, *twilight violet*, *avocado green*, *brown* and *orange*, respectively. The *bar* at the *bottom* of the figure denotes distance

**Fig. 3:**
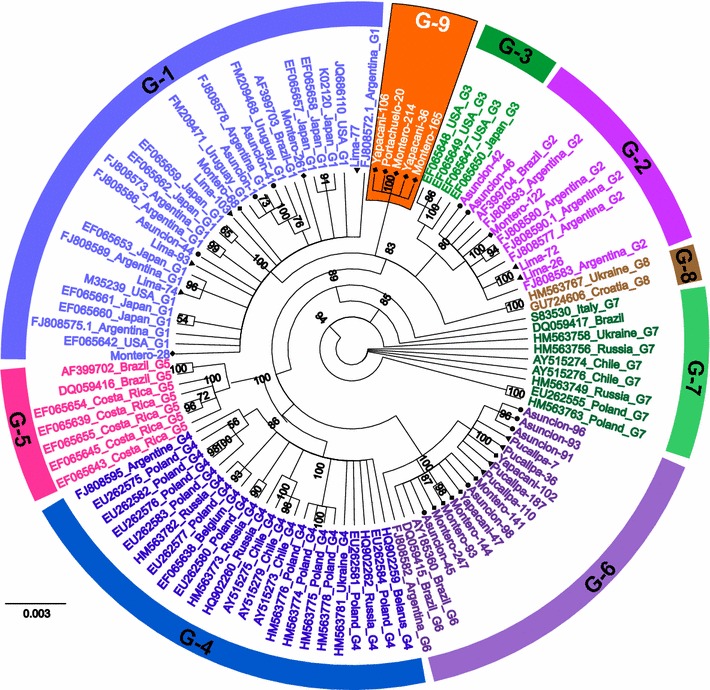
Bayesian phylogenetic tree of BLV *env* partial sequences from different geographical locations worldwide. A Bayesian phylogenetic tree was constructed from 35 distinct 475 bp BLV *env* sequences generated in this study, together with 74 sequences from known BLV strains, representing eight different BLV genotypes from other South American countries, including Argentina, Brazil, Chile and Uruguay, and other parts of the world. Strains in the tree are indicated by accession number and country of origin. South American BLV strains from this study are marked by country of origin as follows: Peru (*filled triangle*), Paraguay (*filled circle*), and Bolivia (*filled diamond*). Genotypes are indicated by *numbers* around the circumference of the figure. Genotypes-1, -2, -3, -4, -5, -6, -7, -8, and -9 are shown in *pastel blue*, *violet*, *sea green*, *blue*, *pink*, *twilight violet*, *avocado green*, *brown* and *orange*, respectively. *Numbers* at nodes indicate posterior probabilities. The *bar* at the *bottom* of the figure denotes distance

Recent phylogenetic studies on the *env* gene of BLV strains isolated worldwide demonstrate that these viruses can be classified into eight genotypes [20–33]. However, no studies of the molecular characteristics of BLV in Bolivia, Paraguay, and Peru have previously been published. Therefore, to gain insight into the degree of genetic variability of BLV strains in South America, partial sequences of *env* gp51 from 131 field strains, representing 30 % of BLV positive samples collected from each country, were amplified. After direct sequencing of these amplicons, because of strains from the same farm having 100 % identity to each other, 475 bp nucleotide sequences of 35 distinct strains among 131, corresponding to nucleotide positions 5090–5564 of the full-length BLV genome (BLV cell line FLK-BLV strain pBLV913, accession number EF600696) [47], were aligned with 74 corresponding sequences from known BLV strains, representing eight different BLV genotypes from additional South American countries (Argentina, Brazil, Chile, and Uruguay) and other parts of the world [27–29, 32]. Phylogenetic trees were then constructed using Maximum likelihood (ML; Fig. 2) and Bayesian Inference (BI; Fig. 3) approaches. The ML and BI trees showed congruent topologies, supported by moderate to high bootstrap values and high posterior probabilities. As shown in Figs. 2 and 3, in contrast to previous studies where only eight BLV strains were detected, both methods of phylogenetic tree construction identified nine sequence clusters, designated genotypes 1-9. All of the BLV strains we collected from Peru were assigned to genotypes-1, -2, and -6. Likewise, the majority of Paraguayan BLV strains clustered into genotypes-1 and -6, with a small number in genotype-2, together with Argentine BLV strains. Interestingly, the BLV strains collected from Bolivia clustered not only into genotypes-1, -2, and -6, together with our Peruvian and Paraguayan strains, but also into a unique clade, which was distinct from the eight previously known BLV genotypes. This result demonstrates that some of our Bolivian BLV strains did not cluster with any others and were highly divergent from the eight known genotypes. This novel genotype displayed a high bootstrap supporting value (92 %) in the ML tree (Fig. 2) and a posterior probability of 100 % using the BI approach (Fig. 3), indicating the presence of new genotype, genotype-9. Furthermore, the genotyping result of reference sequences from Argentine, Brazil, Chile and Uruguay in this study is in agreement with previous studies [20, 23, 24, 26, 31, 32]. Argentine BLV strains were mainly assigned into genotypes-1 and -2, with relatively few samples grouped into genotypes-4 and -6. Reference sequences of Brazilian BLV strains demonstrated a wider variety of BLV genotypes, consisting of genotypes-1, -2, -5, -6, and -7. Known Chilean BLV strains were limited to genotypes-4 and -7. In addition, strains from Uruguay were assigned to genotype-1 only. Thus, our results demonstrate that BLV strains assigned to at least seven genotypes (genotypes-1, -2, -4, -5, -6, and -7, plus the novel genotype-9) are circulating in South America.

### Whole genome sequencing and sequence comparison of strains with genotypes-1, -2, and -6, and a novel genotype-9

To compare the divergence of the BLV whole genome, overlapping genomic fragments covering the complete BLV genomic sequence were amplified from strains grouped into the novel genotype-9 and those in other genotypes, namely genotype-1, -2 and -6, as determined by dual phylogenetic analyses of partial *env* gp51 sequences. Twenty-five samples, including one from Peru, seven from Paraguay, and 17 from Bolivia, were amplified by PCR (Additional file 1: Figure S1) and the amplicons were pooled to construct a DNA library for multiplex sequencing on the MiSeq system (Illumina). Short-read sequences were assembled using three BLV reference sequences (Accession numbers; EF600696, FJ914764, and AF033818) and the average depth of genome coverage was between 143 and 1558 (Additional file 2: Table S1). Moreover, results of analyses of whole genome sequences were 100 % concordant with those of partial *env* sequences produced by Sanger sequencing. Comparative analyses indicated that whole genome sequences obtained from the 25 samples showed 98.29–100 % homology with each other, while they were 95.63–99.42 % homologous to the FLK-BLV strain pBLV913 sequence.

We next calculated the average substitution rate of paired sequences for each gene between the 25 novel complete BLV provirus genomes and those of eight previously reported BLV whole genomes. The numbers of nucleotide and amino acid substitutions per site were estimated using the Jukes and Cantor, and *p*-distance models, respectively. Table 2 indicates the degree of variation in each of the structural genes for a total of 33 BLV strains, including *gag* (*p15*, *p12*, and *p24*), *pro*, *pol*, and *env* (*gp30* and *gp51*), non-structural genes, including the *tax*, *rex*, *R3*, and *G4*, and LTRs. The average nucleotide substitution rate for the whole BLV genome was 0.023 per site. Comparisons between each gene revealed that *G4*, *rex*, and the LTR genes had the lowest average nucleotide substitution rates. By contrast, the genes *R3*, *p24*, and *gp30* revealed the highest average rate of nucleotide substitution. Comparison of amino acid substitution rates between BLV proteins revealed that *tax* and *R3* were the most polymorphic, whereas *p24* was significantly conserved.

**Table 2:** Nucleotide and amino acid substitution rate for 25 novel BLV complete provirus and eight known BLV whole genome

	LTR	p15	p24	p12	pro	pol	gp51	gp30	tax	rex	R3	G4	All
Nucleotide^a^	0.013	0.023	0.030	0.022	0.025	0.023	0.023	0.027	0.023	0.016	0.027	0.013	0.023
Amino-acid^b^	–	0.020	0.006	0.012	0.021	0.014	0.018	0.018	0.053	0.030	0.057	0.028	–

^a^Average nucleotide substitution per site calculated using the Jukes-Canter model

^b^Average amino acid substitution per site calculated using the p-distance model

### Confirmation of the existence of a novel BLV genotype, genotype-9, by phylogenetic analysis of whole genome sequences

**Fig. 4:**
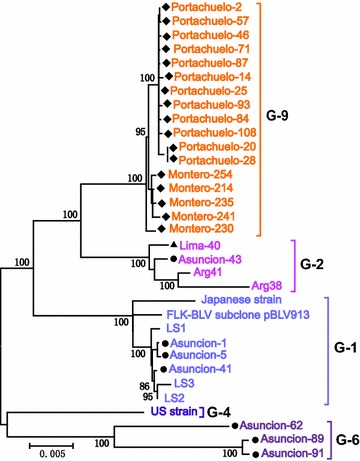
Maximum likelihood phylogenetic tree constructed using complete BLV genomic sequences. A maximum likelihood phylogenetic tree was constructed from complete genomic sequences of BLV strains from a total of 25 samples (submitted to GenBank nucleotide sequence database and assigned accession numbers LC080664, LC080670, LC080669, LC080671, LC080673, LC080665, LC080667, LC080674, LC080672, LC080675, LC080666, LC080668, LC080663, LC080659, LC080661, LC080662, LC080660, LC080654, LC080655, LC080651, LC080652, LC080653, LC080656, LC080657, LC080658) including one sample from Peru, seven from Paraguay and 17 from Bolivia, together with eight reference sequences obtained from the GenBank nucleotide sequence database (whole genomes of FLK-BLV pBLV913, LS1, LS2, LS3, Arg41, Arg38, Japan, and USA with accession numbers EF600696, HE967301, HE967302, HE967303, FJ914764, AF257515, K02120, and AF033818, respectively). Gaps/missing data were treated as partial deletions and, finally, 8374 of 8728 sites were used for the calculation. One thousand replications were performed for calculation of bootstrap values (indicated on the tree). Strains from this study are indicated in the tree by the sample ID together with the location of collection. Reference sequences in the tree are indicated by strain name. Genotypes are indicated by *numbers* to the *right* of the figure. South American BLV strains in this study are marked according to country of isolation as follows: Peru (*filled triangle*), Paraguay (*filled circle*), and Bolivia (*filled diamond*). Genotypes-1, -2, -4, -6, and -9 are shown in *pastel blue*, *violet*, *blue*, *twilight violet* and *orange*, respectively. The *bar* at the *bottom* of the figure denotes distance

Twenty-five novel and eight previously reported BLV whole genome sequences, which were assigned to genotypes-1, -2 and -4, were used to construct a ML phylogenetic tree (Fig. 4). Since the whole genome sequences of genotypes-3, -5, -6 and -8 are not available, we sequenced, for the first time, the whole genome sequence of genotypes-6 and included in phylogenetic analysis. The tree clearly demonstrates stratification of BLV genotypes, including genotypes-1, -2, -4, and -6, and the novel genotype-9, into separate clades (Bootstrap values 100 % for every clade). Thus, this analysis provides evidence for the existence of a novel genotype-9, since the results were identical to those obtained using partial *env* gp51 sequences. However, in this tree based on BLV whole genome sequences, genotype-9 sequences from Portachuelo and Montero were grouped in separate branches. This result indicates at least two different strains within the novel genotype-9 in Bolivia.

### Variations in structural and non-structural genes in 16 distinct novel BLV whole genome sequences from Peru, Paraguay, and Bolivia

**Fig. 5:**
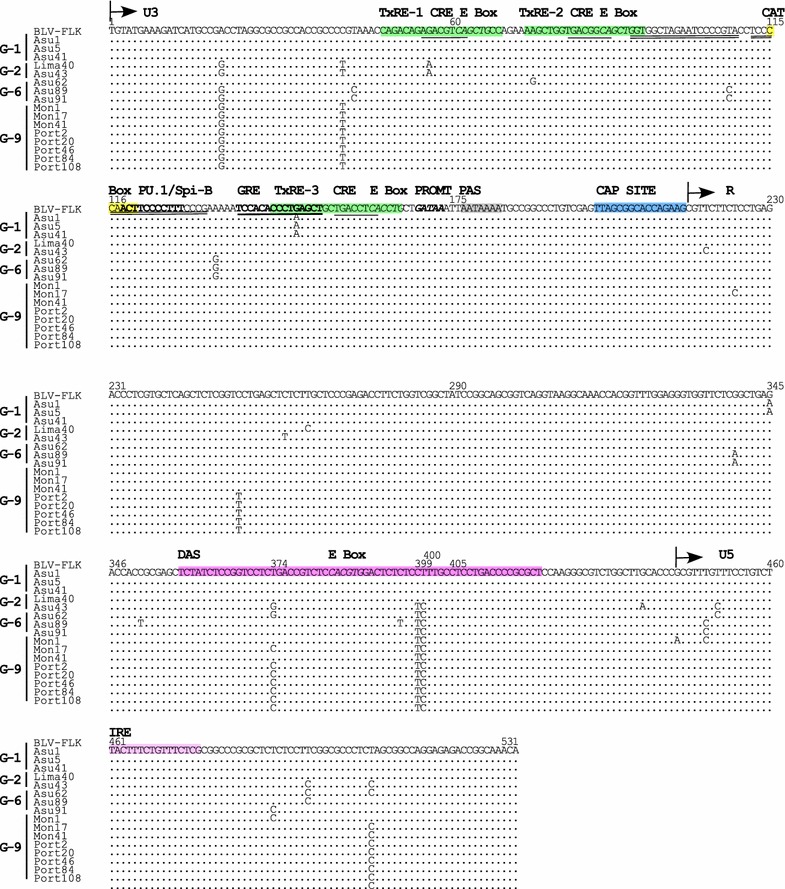
Alignment of BLV 5′LTR nucleotide sequences. Alignment of 5′LTR nucleotide sequences from a total of 16 South American BLV strains, including one, seven, and eight samples from Peru, Paraguay, and Bolivia, respectively, together with that of the FLK-BLV strain pBLV913. Strains from South America are shown by location of sample collection and sample ID. Identity with the FLK-BLV strain is indicated by *dots*. The U3, R, and U5 regions are indicated above the alignment. The three TxRE enhancer regions are shown in *green*, cyclic AMP-responsive element (CRE) sequences are *underlined*, and E-Box sequences are shown in *italics*. Binding sites for PU.1/Spi-B are shown in *bold*. The glucocorticoid responsive element (GRE) binding site is shown in *bold* and *underlined*. Nuclear factor κB (NF-κB) binding sites are shown *double underlined*. The CAT box and GATAA box promoters (PROMT) sequences are indicated in *yellow* and in *bold italics*, respectively. The polyadenylation site (PAS) is shown in *grey* and the CAP site is shown in *blue*. The downstream activator sequence (DAS) and the interferon regulatory factor (IRF) binding sites are shown in *pink* and *light pink*, respectively

**Fig. 6:**
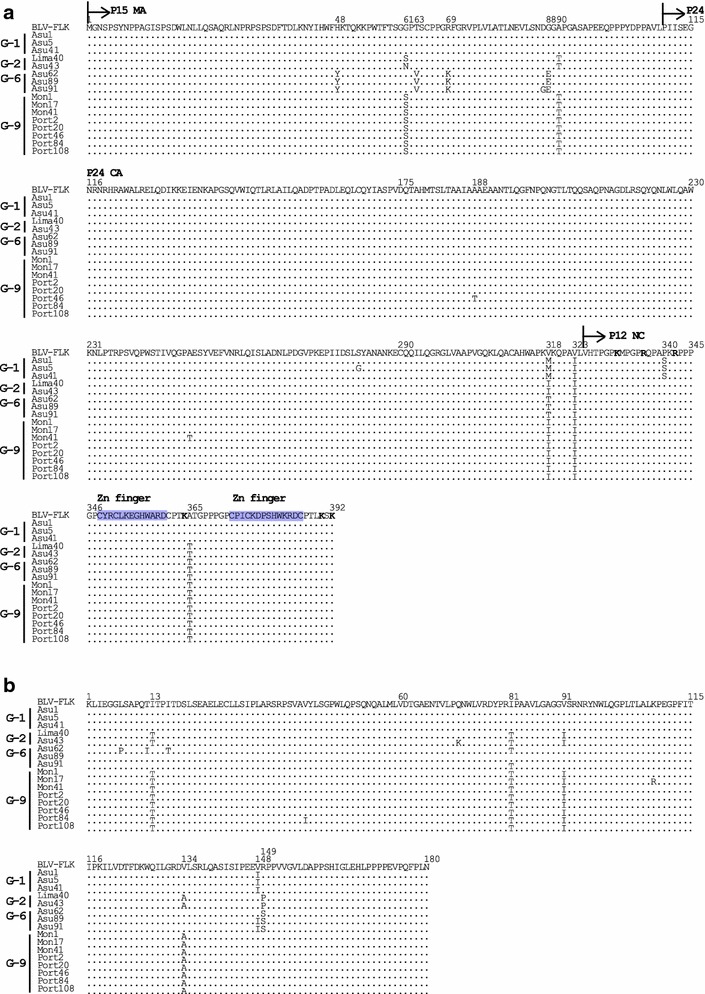
Alignment of amino acid sequences of BLV Gag and Protease (Pro) proteins. Alignment of amino acid residues corresponding to Gag polyprotein (**a**) and Pro protein (**b**) from a total of 16 South American BLV strains including one sample from Peru, seven from Paraguay, and eight from Bolivia, together with that of FLK-BLV strain pBLV913. *Arrows* designate the putative Matrix (p15MA), Capsid (p24CA) and Nucleocapsid (p12NC) protein sequences. Zinc finger domains are shown in *purple*. NC residues in *boldface* indicate basic residues. Identity with the FLK-BLV strain is indicated by *dots*

**Fig. 7:**
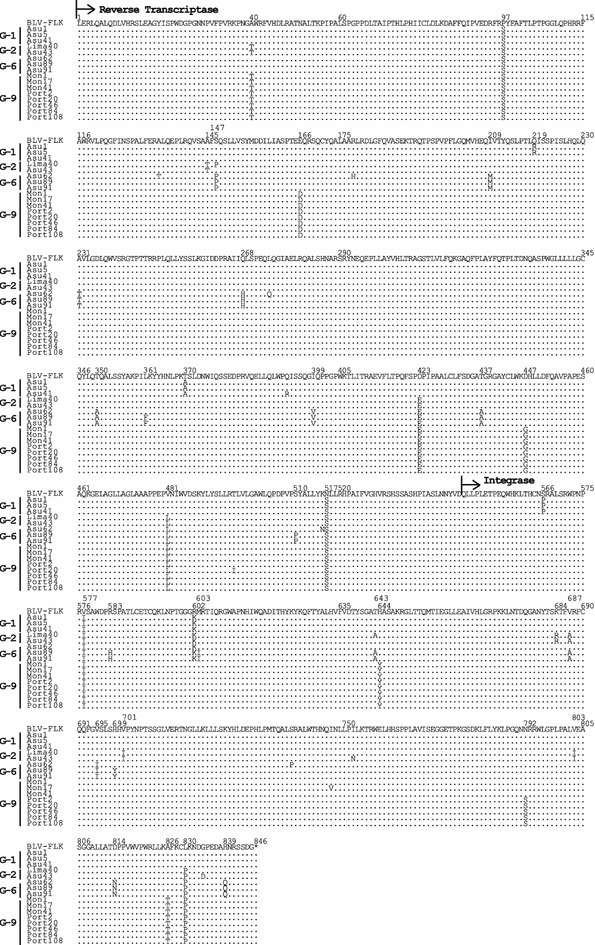
Alignment of amino acid sequences of BLV polymerase (Pol) proteins. Alignment of amino acid residues corresponding to Pol proteins of a total of 16 South American BLV Strains, including one sample from Peru, seven from Paraguay and eight from Bolivia, together with that of the FLK-BLV strain pBLV913. *Arrows* designate the putative reverse transcriptase (RT) and integrase (IN) regions. Identity with the FLK-BLV strain is indicated by *dots*

**Fig. 8:**
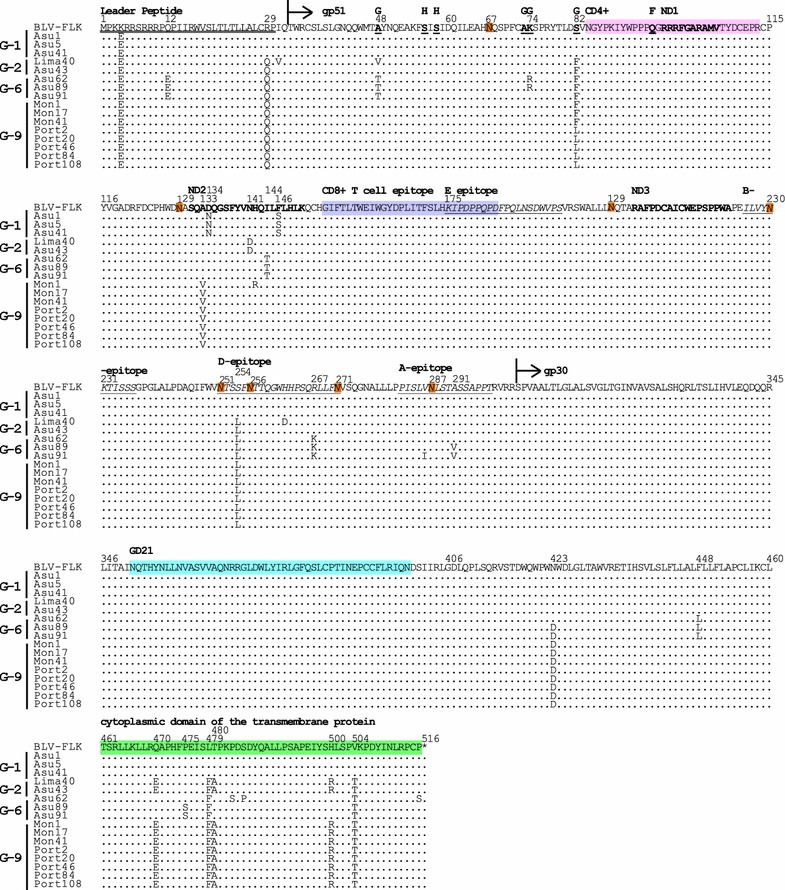
Alignment of amino acid sequences of BLV Envelope (Env) proteins. Alignment of amino acid residues corresponding to Env proteins from a total of 16 South American BLV strains, including one sample from Peru, seven from Paraguay and eight from Bolivia, together with that of the FLK-BLV strain pBLV913. *Arrows* designate the putative gp51 (SU) and gp30 (TM) proteins. The leader peptide is *double underlined*. The conformational epitopes, *F*, *G* and *H* are indicated in *bold* and *underlined*. Linear epitopes, *A*, *B*, *D* and *E* are shown in *italics* and *underlined*. Amino acids involved in neutralization domains are shown in *bold*. The CD4^+^ T and CD8^+^ T epitopes are shown in *pink* and *purple*, respectively. GD21 is shown in *blue*. The cytoplasmic domain is indicated in *green*. Identity with the FLK-BLV strain is indicated by *dots*. The eight *N*-glycosylation sites are shown in *orange box*

**Fig. 9:**
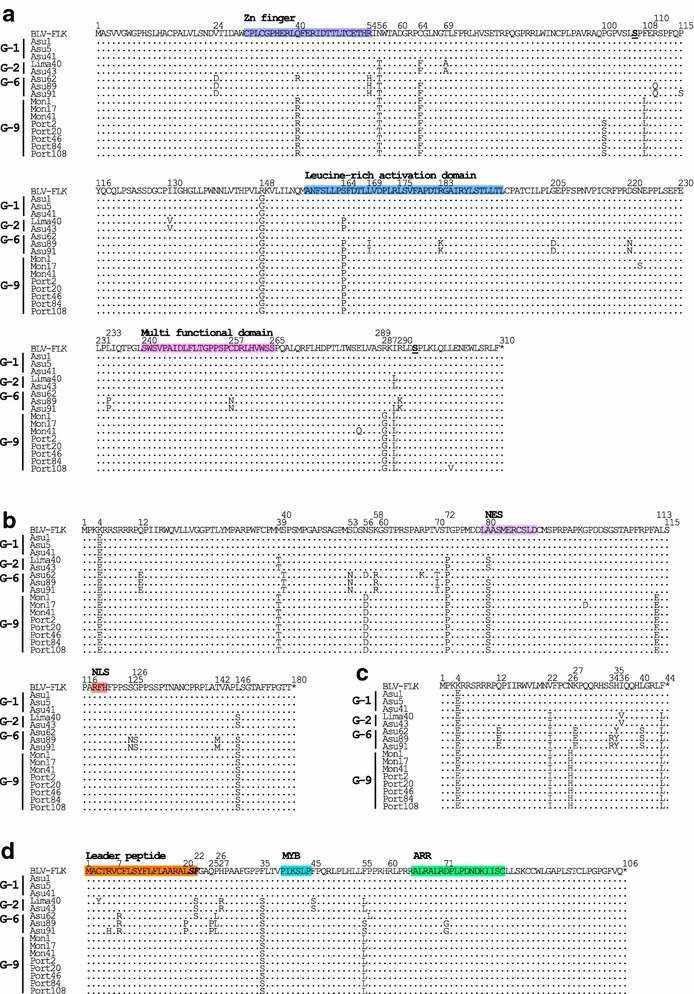
Alignment of amino acid sequences of the BLV regulatory and accessory proteins. Alignment of amino acid residues corresponding to the regulatory proteins Tax (**a**) and Rex (**b**), and the accessory proteins R3 (**c**) and G4 (**d**) from a total of 16 South American BLV strains including one sample from Peru, seven from Paraguay and eight from Bolivia, together with that of the FLK-BLV strain pBLV913. Identity with the FLK-BLV strain is indicated by *dots*. **a** A putative zinc finger, leucine-rich activation, and multiple functional domains in the Tax protein are shown in *purple*, *blue*, and *pink*, respectively. Sites of phosphorylation in the Tax protein are indicated in *bold*, *italics*, and *underlined*. **b** The nuclear export signal (NES) and the nuclear localization signal (NLS) in the Rex protein are shown in *light purple* and *light red*, respectively. **d** The leader peptide, the myb-like motif (MYB), and the arginine-rich nucleus targeting RNA-binding region (ARR) are shown in *orange*, *blue*, and *green*, respectively. Two putative cellular protease cleavage sites are in *italics* and *bold*. Termination codons are shown by an *asterisk*

**Fig. 10:**
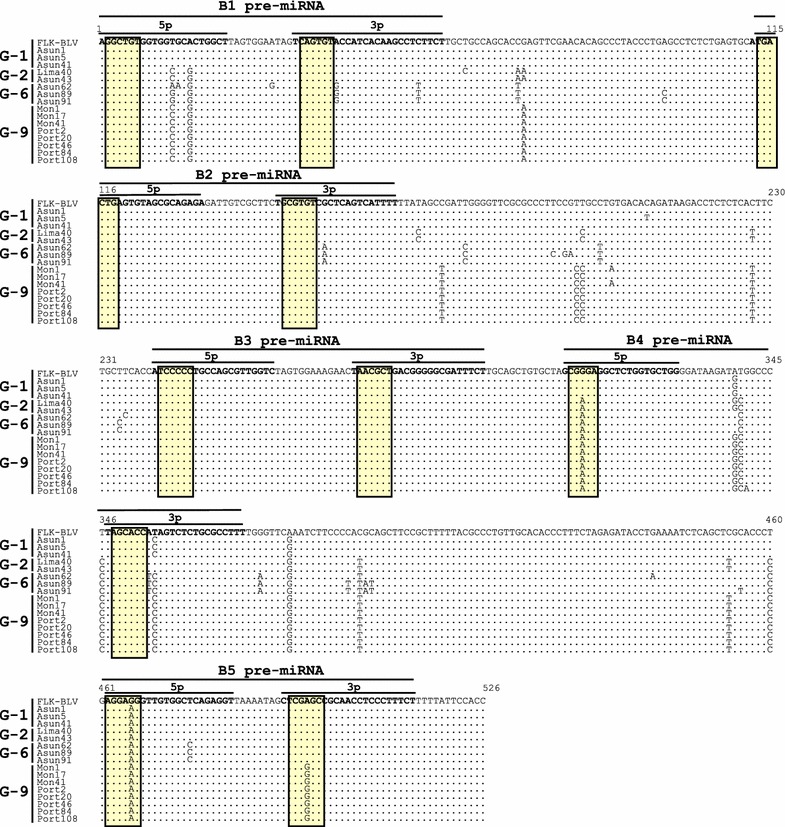
Alignment of nucleotide sequences of BLV miRNAs. Alignments of the nucleotide sequences of miRNAs encoded by BLV strains from 16 distinctive South American samples, including one from Peru, seven from Paraguay, and 8 from Bolivia, together with eight reference sequences from GenBank. miRNA seed regions are shown in *yellow*

To compare the new BLV genotype-9 with previously reported South American genotypes-1, -2, and -6, whole nucleotide or predicted amino acid sequences, including *gag* (*p15*, *p12*, and *p24*), *pro*, *pol*, *env* (*gp30* and *gp51*), non-structural genes, including *tax*, *rex*, *R3*, *G4*, LTRs and miRNAs of 16 distinct strains, selected from 25 unique strains were aligned, using the FLK-BLV strain pBLV913 as a reference sequence (Figs. 5, 6, 7, 8, 9, 10).

BLV transcription is initiated at the U3-R junction of the 5′-LTR by the Tax protein. Transactivation requires the presence of three 21 bp enhancer elements (Tax-responsive elements, TxRE) located in the U3 region of the 5′-LTR. Each TxRE contains a cyclic AMP-responsive element (CRE) and an E-box sequence, which overlaps the CRE motif. The U3 region also contains a PU.1/Spi-B binding site and a glucocorticoid responsive element (GRE). In addition, BLV expression is regulated by 5′-LTR sequences downstream of the transcription initiation site (DAS) at the 3′end of the R region and an interferon regulatory factor (IRF) binding site in the U5 region. Comparison of the 16 LTR sequences of four South American genotypes, genotypes-1, -2, -6 and -9, with that of FLK-BLV strain pBLV913 revealed that these entire regions, including the CRE motifs, E Boxes, CAT Boxes, polyadenylation sites (PAS), and IRF binding sites, were well conserved overall (Fig. 5). Interestingly, three nucleotide changes were observed downstream of the DAS, at residues 374, 399, and 400 in sequences of genotype-9 strains and strains of other genotypes. There are also some nucleotide changes outside of the functional motifs (Fig. 5).

Gag is a polyprotein precursor made up of p15 MA, p24 CA, and p12 NC proteins (Fig. 6a). All of these polyprotein regions were highly conserved among the aligned sequences. For the p15 MA protein, sequences of three strains assigned to BLV genotype-1 were identical to that of pBLV913, whereas BLV strains clustered into genotypes-2, -6, and -9 had amino acid substitutions at residues 48, 61, 63, 69, 88 and 90 (Fig. 6a). The p24 CA protein region was also well conserved overall, with substitutions detected at residues 318 (V318 M/I/T), and 323 (V323I) (Fig. 6a). The p12 NC protein is constituted of a region rich in basic amino acid residues and zinc binding domains involved in RNA packaging, both of which are very well conserved. A study by Wang et al. concluded that substitutions affecting either the basic amino acid residues or the zinc finger domains might lead to a reduction in RNA packaging [48]. In our study, all basic residues and Zn finger domains were conserved in the BLV strains analyzed (Fig. 6a), but a substitution from Proline to Serine at amino acid residue 340 (P340S) was detected in the p12 NC protein region of South American genotype-1 strains. This substitution has previously been reported in genotype-1 BLV strains LS1, LS2, and SL3 [49]. In addition, another substitution at residue 365 (A365T) was detected in strains of genotypes-2, -6, and -9. However, the functional consequences of these substitutions are unknown.

The BLV Pro protein is an aspartic protease with a function in *gag* processing and thus virion maturation [49]. As shown in Fig. 6b, although substitutions at residues 13, 81, 91, 134, 148 and 149 were detected among the Pro protein sequences of South American strains, this region was generally well conserved and no new genotype-specific substitutions were detected.

The deduced BLV RT and IN amino acid sequences encoded by the *pol* gene of all of 16 South American BLV strains were also highly conserved (Fig. 7). The polymerase (Pol) amino acid sequences of the 16 samples were similar to the reference sequence; however, interesting and important findings included substitutions of glutamic acid to aspartic acid (E166D) and aspartic acid to glycine (D447G) in the RT region, and from histidine to tyrosine (H644Y) and alanine to threonine (A826T) in the IN region, observed only in all sequences of strains assigned to the new BLV genotype-9 (Fig. 7). In addition, an asparagine to serine substitution (A792S) was detected only in the IN protein sequence of all 12 samples collected from Portachuelo, which were grouped into genotype-9, but this substitution was not detected in other genotype-9 strains, indicating that particular amino acid changes could be limited to certain geographic regions (Fig. 7). In addition, nine substitutions at residues 209, 231, 268, 350, 399, 437, 695, 814, and 839 were found in the Pol protein region of three sequences from Bolivian strains clustered into genotype-6.

The sequences encoding the Env leader peptide and gp30 protein were highly conserved among all of our 16 South American BLV strains, as were those of gp51 proteins (Fig. 8). Comparisons of conformational (F, G, and H) and linear (A, B, D, and E) epitopes demonstrated that A, B, E, F, and H were conserved, while the G-epitope at residues 48, 74 and 82, and D-epitope at residues 254 and 267 showed divergence (Fig. 8). The K74R substitution has previously been described in *env* gp51 partial sequence deposited in the GenBank database [32]. The S82F substitution was present in sequences from strains grouped to genotypes-2, -6, and -9. Interestingly, the S82L substitution was detected only in all 12 sequences of Portachuelo-derived strains grouped in genotype-9. Of the neutralizing domains, the first and third were conserved, while the second demonstrated some genotype-specific substitutions (Fig. 8). Genotype-1 samples collected from Paraguay showed substitutions of aspartic acid to asparagine at residue 134 (D134N) and of phenylalanine to serine at residue 146 (F146S), which have previously been observed in some *env* gp51 partial sequences isolated from Uruguay and Brazil [33]. In addition, BLV strains assigned into genotypes-2 and -6 also showed specific substitutions, N141D and I144T, respectively, in the second neutralizing domain. Interestingly, one significant substitution was observed at position 133 of the gp51 protein, with alanine (A) substituted for valine (V) in all BLV genotype-9 strains, in agreement with results obtained by direct sequencing of the partial gp51 *env* region. Furthermore,both gp51 partial sequences and full genome sequences showed the conservation of all of the eight N-linked glycosylation sites. In the gp30 protein, the fusion peptide and GD 21 were highly conserved, while cytoplasmic domain of the transmembrane protein showed divergence with some substitutions, including Q470E, L479F, T480A, H500R, and V504T, commonly detected in South American BLV strains of various genotypes (Fig. 8).

The functional domains of the BLV Tax protein, which regulates BLV expression, include a putative zinc finger motif (residues 30–53) [2, 3], a leucine-rich activation domain (residues 157–197) [50], two phosphorylation sites (residues 106 and 293) [51], and a multi functional domain (residues 240–265) [2, 52–57]. These domains were assigned in the four South American genotypes, -1, -2, -6, and -9, and pBLV943, and compared between the 16 distinct strains. In the deduced amino acid alignments of the three functional domains of Tax, substitutions at residue 40 (Q40R) in the zinc finger domain, residue 164 (S164P), residue 169 (L169I) and residue 183 (R183 K) in the leucine-rich activation domain, and residue 257 (C257 N) in the multi functional domain were detected in South American BLV strains (Fig. 9a). By contrast, the two phosphorylation sites were conserved in all South American BLV strains included in this study. The most important findings in the alignment of the Tax protein were substitutions at residue 108 (F108L), detected only in sequences of all of the new South American genotype-9 BLV strains, and a substitution at residue 100 (P100S), observed only in samples collected from Portachuelo, indicating genotype-9 and/or Portachuelo region specific mutations in Tax.

Another BLV regulatory protein, Rex, is associated with nuclear pores and harbors a nuclear localization signal (NLS) and a nuclear export signal (NES) required for RNA-binding and nuclear localization (Fig. 9b). Alignment of deduced amino acid sequences from the 16 South American BLV strains showed that the NLS domain was well conserved, while one substitution (A80S) was detected in the NES of the majority of samples. In addition, other mutations, including M39T, T72P, and L146S, were commonly detected in BLV strains with genotypes-2, -6, and -9, and were also present in sequences deposited in the GenBank database. Five substitutions at residues 12, 40, 53, 58, and 70 were detected only in BLV strains of genotype-6. Of interest, one substitution at residue 113 (A113E) of the Rex protein was observed only in all genotype-9 strain sequences.

The amino acid sequence of the accessory protein, G4, includes an amino terminal stretch of hydrophobic residues (amino acids 1–24) followed by a potential proteolytic cleavage site, a myb-like motif (MYB; amino acids 39–44) and an arginine-rich region (ARR; amino acids 58–72) located in the middle of the protein [58] (Fig. 9d). The MYB and ARR of G4 were well conserved; however, a substitution from phenylalanine to serine at residue 22 was detected in one of the two cleavage sites in three South American BLV strains of genotypes-2 and -6. The functional effect of this F22S substitution is unknown. Other point mutations at residues 7, 25, 26, 27, 35, 45, 55, and 71 were found in some South American BLV strains.

By contrast, as shown in Fig. 9c, the alignment of the deduced amino acid sequences of another accessory protein, R3, demonstrated that a substitution at residue 26 (N26H) was detected only in the sequences of all genotype-9 BLV strains. In addition, four substitutions, Q12E, K27E, H35Y and L40S, were observed only in three South American BLV strains clustered into genotype-6, indicating genotype-specific mutations in the R3 protein.

BLV-encoded micro RNAs (miRNAs) might possibly play potential roles in the regulation of gene expression and in tumor development [14, 15]. In this study, we also obtained sequences of ten unique BLV miRNAs derived from five predicted miRNA precursor hairpins, BLV-premiR-B1 to -B5 (Fig. 10), corresponding to those included in previous studies [14, 15]. All of the ten miRNA sequences were well conserved, with BLV-miRNA-B2-5p, BLV-miRNA-B3-5p, and BLV-miRNA-B3-3p conserved in all four South American BLV genotypes included in the analysis. In addition, the seed sequences of the predominant arms of seven of the ten miRNAs were identical across all examined BLV strains. However, all genotype-9 BLV strains had an A to G nucleotide change in the seed sequence of BLV-miRNA-B5-3p. In addition, nucleotide substitutions of A to G and C to T in BLV-miRNA-B1-3p, G to A in BLV-miRNA-B2-3p, A to T in BLV-miRNA-B4-3p, and T to C in BLV-miRNA-B5-5p were detected only in BLV genotype-6 strains.

### BLV genotype-9 specific mutations

**Fig. 11:**
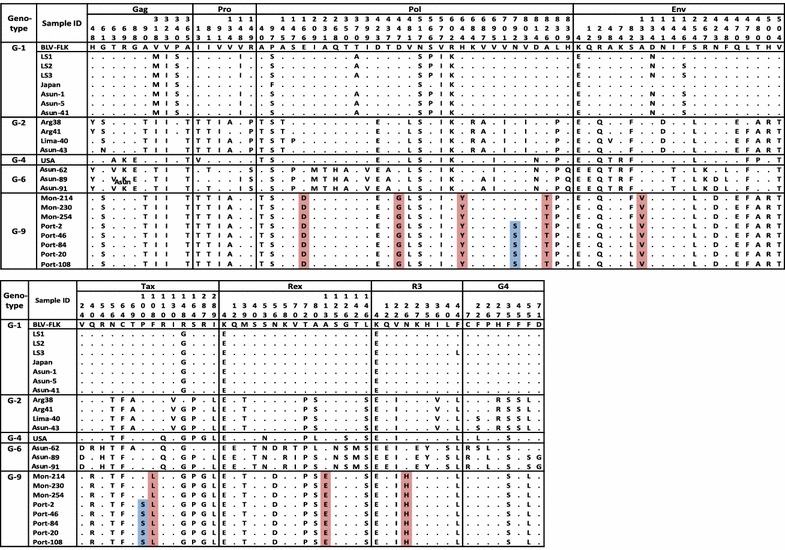
Summary of amino acid substitutions in sixteen novel and eight known BLV whole genome sequences. Amino acid substitutions identified in at least three samples are shown. South American BLV strains from this study are marked according to the country of isolation as follows: Peru (*filled triangle*), Paraguay (*filled circle*), and Bolivia (*filled diamond*). Genotypes are indicated by *numbers* in the figure. *Light red color* indicates genotype-9-specific mutations detected in all samples, and *light blue* color indicates genotype-9-specific mutations detected only samples collected from Portachuelo in Bolivia

Sixteen distinct novel BLV complete provirus genome sequences from Peru, Paraguay, and Bolivia were aligned with eight previously reported BLV whole genome sequences, including FLK-BLV pBLV913 [47], strains LS1-LS3 from Uruguay [48], strains Arg41and Arg38 from Argentina [59, 60], and two strains from Japan [4] and the USA. As summarized in Fig. 11, ten unique amino acid substitutions were observed in genotype-9 BLV strains as follows: (1) Two substitutions, E166D and D447G, in the Pol (RT) region; two substitutions, H644Y and A826T, in Pol (IN) region; and one substitution at residue 792 of the Pol (IN) region, restricted to only sequences of all 12 samples collected from Portachuelo, but not in other genotype-9 strains. (2) In the Env (gp51) protein, one significant substitution, A133 V, was observed in all BLV genotype-9 strains and not detected in eight known whole BLV genome sequences. Interestingly, another substitution in the Env (gp51) protein, S82L, was detected only in all of the 12 Portachuelo strain sequences of genotype-9, but not in other genotype-9 sequences. (3) In the regulatory proteins Tax and Rex, substitutions at residue 108 (F108L) of Tax and residue 113 (A113E) of Rex were detected only in genotype-9 strains, but not in other strains or the eight known BLV genome sequences. In addition, a substitution at residue 100 (P100S) of the Tax protein was observed only in samples collected from Portachuelo. (4) Likewise, a substitution at residue 26 (N26H) in the R3 accessory protein was detected only in the sequences of all genotype-9 BLV strains, but not in other strains or known BLV genome sequences. These ten substitutions were first detected in the sequences of the new genotype-9 strains identified in this study and the impact of these substitutions on the function of the proteins will require further study. In addition, 15, 7, and 22 unique amino acid substitutions were observed in BLV strains of genotypes-1, -2, and -6, respectively (Fig. 11).

## Discussion

We draw four major conclusions from the results of this study of BLV in South American cattle. First, we identified a novel BLV genotype, genotype-9, in samples from two Bolivian provinces, Montero and Portachuelo, by phylogenetic analyses of partial *env* gp51 and whole genome sequences. Recent phylogenetic studies using BLV *env* gene sequences from strains isolated worldwide have classified the virus into eight genotypes [20–33]. Thus, the detection of genotype-9 is a novel finding of this study. Second, ML phylogenetic analysis of BLV whole genome sequences showed that genotype-9 sequences derived from Portachuelo were distinct from those collected in Montero. Importantly, this result was obtained from BLV whole genome sequence data, but was not apparent from analyses of partial *env* gp51 sequences. Third, current research provides the new information of the full genome sequences of a novel genotype-9 and genotype-6 (partially known *env* sequences). Fourth, our data provides updated information about the spread of BLV infection in South American countries and demonstrates a high rate of BLV infection in five countries: Peru, Paraguay, Bolivia, Argentina, and Chile. This information will be important for the control of BLV infection in South America, and the data regarding the prevalence of BLV in Bolivia and Paraguay is unique to this study.

The most interesting data in this study are the phylogenetic results of the existence of genotype-9 confirmed by the ML method using 25 novel and eight previously reported BLV whole genome sequences (Fig. 4), which was in agreement with the phylogenetic analyses of 109 partial *env* gp51 sequences obtained by two independent methods (Figs. 2, 3). Notably, the bootstrap value of the genotype-9 cluster was remarkably increased in the ML phylogenetic tree constructed from complete genome sequences (bootstrap value = 100 %), compared to that constructed using partial *env* sequences (bootstrap value = 92 %). Therefore, the NGS analysis provided strong confirmatory evidence that genotype-9 is separate from the other eight genotypes.

As shown in Fig. 11, our comparison of BLV amino acid sequences showed that 10 unique amino acid substitutions were observed in the Pol, Env, Tax, Rex, and R3 regions of genotype-9 BLV strains. Importantly, two of these substitutions, including N792L in the *Pol* region and P100S in *Tax*, were observed only in samples collected from Portachuelo assigned to genotype-9, indicating the presence of at least two distinct genotype-9 strains in Bolivia. This comparison also revealed that 15, 7, and 22 unique amino acid substitutions are encoded by BLV genotypes-1, -2, and -6, respectively. This result was confirmed by ML phylogenetic analysis with BLV whole genome sequences, which also divided Bolivian genotype-9 sequences into at least two strains.

Phylogenetic analyses of the *env* gp51 gene also suggested the possibility that genotype-6 may be divided into three sub-genotypes, including clades for (1) the FJ808582/Argentina strain, (2) the DQ059415/Brazil and AY185360/Brazil strains, and (3) our newly-identified sequences, including Paraguay-91. By whole genome analysis, Paraguay-62, which was assigned to clade (2) in the gp51 *env* analysis, was clearly divided from Paraguay-91, with a bootstrap value of 100 %. Our previous work has also suggested that genotype-6 may be divided into three subgroups: G-6a, G-6b and G-6c [30]. Therefore, these two branches, including Paraguay-62, and Paraguay-89 and -91 may be designated as separate genotypes when additional whole genome sequences of genotype-6 are accumulated and analyzed in future.

This study revealed a widespread distribution and remarkably high levels of BLV in five South American countries, including Peru, Chile, Argentina, Paraguay, and Bolivia. In Peru, 139 of 328 cattle samples were positive for BLV provirus, with a prevalence rate of up to 58.6 %. Of Peruvian samples, 58.6 % of those from Holstein cattle in Lima were BLV positive, whereas 40.0 % of samples from Pucallpa gave positive results. Our results were consistent with those of Ch [44], who demonstrated that BLV infection is widespread in Peru; however, the percentages of BLV positive samples found in the Peruvian areas of Lima and Pucallpa were higher in our study than those reported by Ch (up to 31.0 %) [44]. In Argentina, we found that 325 of 420 samples were BLV positive, with an overall prevalence of 77.4 % at individual level and up to 90.9 % at the herd level. These results are consistent with previous reports demonstrating that BLV is widespread in Argentina [26, 31, 41, 42, 61]. Argentine samples were collected from farms in Buenos Aires, where a high individual BLV prevalence was reported by Trono et al. [43] and our results were broadly consistent with those of Trono et al.; however, we detected a higher BLV prevalence at the herd level, since Trono et al. reported infection levels of 84 % [43]. In addition, at the individual level, the percentage (77.4 %) of BLV positive Argentine samples in our study was slightly higher than that reported by Monti et al. (70 %) [26], indicating a possible increased prevalence of BLV in Argentina. Compared to Peru and Argentine, relatively few BLV positive samples were identified from Chile. However, from the distribution of BLV presented in Table 1, we can see that more breeds and a wider geographical area were involved in BLV infection in Chile, indicating a tendency towards widespread infection, in agreement with a previous study of BLV prevalence in this country [24]. By contrast, over 50 % (76/139) of screened samples from Paraguay were BLV positive and all infected breeds in Paraguay demonstrated a high level of BLV prevalence, ranging from 28.6 to 71.4 % in the Holstein breed, with 36.4 % in Brown Swiss cattle. In Bolivia, 156 of 507 samples were BLV positive and the BLV infection level differed from farm to farm. There are no previous data detailing the prevalence of BLV in Paraguay and Bolivia, and our result is the first report confirming the presence of BLV in these countries.

Based on sequencing results from the BLV provirus, the predicted amino acid sequences of the partial and full gp51 *env* gene from Peruvian, Paraguayan, and Bolivian BLV strains were highly conserved. Amino acid substitutions in the gp51 protein were mostly located in the second neutralization domain, which is consistent with previous studies [32, 33]. In this study, only two distinct genotype-1 BLV strains from Paraguay had amino acid changes at positions 134 (D134N) and 146 (F146S) of the second neutralizing domain. These two substitutions were also observed in Brazilian and Uruguayan BLV genotype-1 strain isolates [33], indicating that these amino acid substitutions exist mainly in South American BLV strains, and suggesting a common origin for these virus strains. Interestingly, Moratorio et al. [33] confirmed that D134N changes the net charge of a loop of the Env protein, indicating a potential impact of this substitution on virus-host interactions. BLV strains grouped into genotype-2 showed a common substitution, N141D. Interestingly, the V191I substitution in the B epitope, observed in only one distinct BLV strain from Peru in this study, has been reported in some Argentine BLV isolates [33]. Most importantly and interestingly, an A133V substitution in the second neutralization domain was observed in only genotype-9 BLV strains. Previous data have demonstrated amino acid changes of alanine to aspartic acid (A to D) or threonine (A to T) [32] at this residue, but there are no previous reports of alanine to valine substitutions, indicating that A133V is a novel finding of this study.

At present, there are eight full-length BLV genome sequences available in the GenBank database. The major limitations of Sanger sequencing compared to Next Generation methods are increased time and cost, and the limited amount of data generated by sequencing runs. The rapid development of NGS (DNA and RNA-seq) has accelerated sequencing analysis of new genomes and transcriptomes of complex organisms, allowing the identification of new virus species integrated within complete or draft genome sequences. Commonly, virus genomes are very compact and the sequences have multiple functions, such as protein encoding (sometimes for multiple proteins), transcription, regulation of host genes, and virus genome packaging. Therefore, identification of complete viral genome sequences is becoming increasingly important. In this study, we identified both BLV partial *env* and complete viral genome sequences from the same samples. Our data indicate that the BLV *env* sequence is a good target for classification of BLV strains, and analysis of *env* sequences was helpful for selection of informative strains to include in the complete genome sequence analysis.

In this study, we sequenced complete genomes of 25 BLV strains, including 17 novel genotype-9 strains. Our results revealed that BLV genomes contain a number of unique genotype specific substitutions not only in the *env* region, but also in the LTR, Gag, Pro, Pol, Tax, Rex, R3, G4, and miRNA encoding regions. The Information about substitutions in viral genomes is important for investigating viral spread worldwide. Therefore, full understanding of the biological functions of these genotype specific substitutions is now essential. By contrast, we also found that BLV genome sequences of strains from different geographic origins, especially the important sites on the regulation of viral replication of BLV, are relatively stable and highly conserved as follows. Three copies of CRE sequences were imperfectly conserved in all strains of each genotype. Merezaki et al. (2001) found that introducing a perfect CRE sequences into TxRE increases the BLV LTR promoter activity, suggesting that imperfect conservation of CREs repress viral expression with escape from the host immune response [62]. Furthermore, deep sequencing results of the current study were consistent with previous result [28] that all of the eight N-linked glycosylation sites are conserved very well. The absence of mutation of N-linked glycosylation sites in naturally infected cattle is explained by the hypothesis as being favorable for the virus to coexist with its host for efficient replication and transmission [63]. Moreover, the two phosphorylation sites on Tax protein, and NES and NLS regions in Rex protein were also conserved well. Thus, the fact that limited sequence variations are compatible with the development of a vaccine [19, 64, 65].

## Conclusion

The present study provides the first evidence of the prevalence of BLV infection among cattle in Paraguay, Peru, and Bolivia, and also confirms the widespread distribution of BLV infection in Argentina and Chile. Our BLV genotyping studies demonstrate that Peruvian and Paraguayan BLV strains are of genotypes-1, -2, and -6. Of interest, our findings indicate that Bolivian BLV strains are clustered into genotypes-1, -2, -6, and -9. The existence of the novel genotype-9 was confirmed by comprehensive phylogenetic analyses of both partial gp51 *env* and full BLV genome sequences.

## Methods

### Experimental samples and extraction of proviral DNA

Blood samples were obtained from a total of 2204 cattle, including 328 from three different farms in Peru, 139 from five farms in Paraguay, 507 from eight farms in Bolivia, 420 from three farms in Argentina, and 810 from 19 farms in Chile (Fig. 1; Table 1). Farms located in main-cattle raising area were chosen for sampling in each country. All animals were handled by veterinarians from RIKEN, Universidad Austral de Chile and LAVET, in strict accordance with good animal practice following the Universidad Austral de Chile Institutional guidelines. This study was approved by the Committee on the Ethics of Animals for Research at the National University of LA PLATA (Certificate date May 26th, 2014) and by the Committee on the Ethics of Animals for Research at Universidad Austral de Chile (Certificate No. 153-2014).

Proviral DNA was extracted from 40 μl of whole blood spotted onto Whatman FTA elute cards (GE Healthcare Japan Corp., Tokyo, Japan), according to the manufacturer’s instructions. The extracted DNA was stored at −20 °C until required for PCR. Cattle were classified as BLV-infected where genomic integration of the BLV provirus was detected.

### Detection of BLV provirus by nested PCR

Each sample was screened for the presence of BLV provirus by the detection of BLV LTR regions using nested PCR, as described previously [30, 66]. Briefly, the first PCR amplification was performed with primers BLTR256F (5′-GAGCTCTCTTGCTCCCGAGAC-3′) and BLTR453R (5′-GAAACAAACGCGGGTGCAAGCCAG-3′). As an internal control, the *BoLA*-*DRA* gene was amplified using primers BDRA488F (5′-ACAACACCCCAAACACCAAT-3′) and BDRA1145R (5′-AGGAAGGGGAGGTAGTGGAA-3′). Five picomoles of each primer, 2 μl of 10× rTaq PCR Buffer (Toyobo, Osaka, Japan), 2 μl of 25 mM MgCl_2_, 2 μl of 2 mM dNTP mix, 0.1 μl of 5U/μl rTaq, and 10.4 μl of nuclease-free water were added to each sample, which was amplified in a final volume of 20 μl for 45 cycles of 94 °C for 30 s, 58 °C for 30 s, and 72 °C for 30 s, and then a final extension step of 4 min. Two μl of Exo-SAP IT (USB Corp., Cleveland, OH, USA) was applied to the initial PCR products and incubated at 37 °C for 15 min, then at 80 °C for 15 min. The initial PCR amplicons were subsequently applied to the second PCR with primers BLTR306F (5′-GTAAGGCAAACCACGGTTT-3′) and BLTR408R (5′-AGGAGGCAAAGGAGAGAGT-3′). Five picomoles of each primer, 2 μl of 10× rTaq PCR Buffer, 2 μl of 25 mM MgCl_2_, 2 μl of 2 mM dNTP mix, 0.1 μl of 5U/μl rTaq, and 11.9 μl of nuclease-free water were added to 1 μl of each purified PCR product. Sterilized water was used as a PCR negative control.

### PCR amplification and sequencing of BLV *env* gene fragments

One hundred and thirty-one BLV field strain samples, consisting of 41, 34, and 56 from Peru, Paraguay, and Bolivia, respectively, representing 30 % of BLV positive samples collected from each country, were randomly chosen for amplification of the BLV *env* gene. The partial BLV *env* gene was amplified by nested PCR. PCR amplification was performed using PrimeSTAR GXL DNA Polymerase (Takara Bio Inc., Otsu, Japan) and the following primers: external forward (5′-ATGCCYAAAGAACGACGG-3′) and external reverse (5′-CGACGGGACTAGGTCTGACCC-3′) described previously [33], and Env_5032_ (5′-TCTGTGCCAAGTCTCCCAGATA-3′) and Env_5608r_ (5′-AACAACAACCTCTGGGAAGGGT-3′) for second-round PCR [67]. The reaction mixture contained 13.5 μl (initial PCR) and 14.5 μl (second PCR) of distilled water, 5 μl of 5× PS GXL Buffer, 2 μl of 2.5 mM dNTP mix, 0.5 μl of PrimeSTAR GXL, and 1 μl of each primer (10 μM). Conditions for PCR amplification were as follows: 98 °C for 2 min, followed by 30 cycles of denaturation at 98 °C for 15 s, annealing at 60 °C for 20 s, and extension at 68 °C for 60 s. The external primers resulted in amplification of a 913 bp DNA fragment, and internal primers amplified a 597 bp fragment of the gp51 region of the *env* gene.

Positive second-round PCR products were purified using Exo-SAP IT (USB Corp., Cleveland, OH, USA) and sequenced on an ABI3730xl DNA Analyzer using an ABI PRISM Big Dye Terminator v 3.1 Ready Reaction Cycle Sequencing Kit (Applied Biosystems, Foster City, CA, USA). Sequences included a 475 bp sequence of the *env* gene, corresponding to nucleotide positions 5090–5564 of the BLV cell line FLK-BLV subclone pBLV913 complete genome (GenBank accession number EF600696) [47]. Editing, alignment, and identification of nucleotide sequences were performed using MEGA 5.1 software [68].

### Phylogenetic analysis of *env* nucleotide sequences

The 131 BLV *env* partial sequences from Peru, Paraguay, and Bolivia were successfully amplified. Because of strains showing homology to each other, 35 distinctive sequences among the 131 were aligned with 74 BLV *env* sequences from GenBank, including sequences from other South American countries and also of BLV isolates from other parts of the world, representative of the eight known BLV genotypes, using MEGA 5.1 software [68]. For robust and accurate phylogenetic analysis of the BLV *env* gp51 partial sequences, phylogenetic trees were constructed using two different algorithms.

First, ML trees were constructed using MEGA 5.1 [68] and Kimura 2-parameter model plus gamma distribution (K2+G) was chosen as the best model for nucleotide substitution [69]. For the ML analysis, the reliability of the phylogenetic relationships was evaluated using nonparametric bootstrap analysis with 1000 replicates.

Next, to confirm the data obtained by ML analysis, BI was performed using MrBayes v.3.2.5 [70] with the evolutionary model set to lset nst = 6, rates = equal [corresponding to the general-time-reversible model (GTL)]. In BI analysis, two runs with four Markov chains were carried out simultaneously for 100,000,000 generations, and the trees were sampled every 100 generations. The first 25 % of the BI trees were discarded as “burn-in”. A consensus tree was constructed from the output file produced in the BI analysis using FigTree v. 1.4.2 (http://tree.bio.ed.ac.uk/software/figtree/).

### PCR amplification of the whole BLV provirus

To gain insight into the genetic differences between the distinct BLV genotypes, PCR amplification of overlapping genomic fragments (Additional file 1: Figure S1) covering the complete BLV genome of 25 South American samples was achieved using the PrimeSTAR GXL DNA Polymerase (Takara Bio Inc., Otsu, Japan) and specific primers designed for this study (Life Technologies Japan Ltd, Tokyo, Japan) (Additional file 1: Figure S1). The 25 μl final reaction mixture contained 5 μl of 5× PrimerSTAR GXL Buffer, 2 μl of 2.5 mM dNTP mix, 1 μl of each primer at a concentration of 10 pmol, 3 μl of template and 0.5 μl of PrimerSTAR GXL DNA Polymerase. The cycles for the PCR amplification were as follows: 98 °C for 2 min, followed by 33 cycles of denaturation at 98 °C for 15 s, annealing at 60 °C for 20–30 s, and extension at 68 °C for 1–6 min (1 min per kilobase), followed by a final extension at 72 °C for 4 min. Each amplicon was quantified using the Qubit dsDNA BR Assay kit (Life Technologies Ltd, Oregon, OR, USA) and four different BLV provirus genome PCR amplicons from each individual were pooled together at an equimolar ratio to a final concentration of 30 ng/μl as the starting material for whole genome sequencing library preparation.

### BLV whole genome library preparation

DNA libraries of the above-mentioned pooled samples were prepared by a transposase-mediated library preparation method, using the SureSelect QXT Library prep for Illumina Multiplexed Sequencing Kit (Agilent Technologies, Santa Clara, CA, USA). Briefly, 30 ng of each pooled sample was treated with 1 μl of the SureSelect QXT enzyme mix ILM and 8.5 μl of QXT buffer, and incubated for 10 min at 45 °C, followed by addition of 16 μl of QXT stop solution, enabling enzymatic fragmentation and addition of adaptors to the ends of fragments in a single reaction. The adaptor-tagged library fragments were purified using Agencourt AMPure XP beads (Beckman Coulter, Inc., Brea, CA, USA) with the NGS Magna Stand Ch YS-Model (NIPPON Genetics Co., Ltd., Tokyo, Japan) according to the manual. Next, each purified adaptor-tagged library was PCR amplified with 1 μl of each of the indexing primers, P5 and P7, added to the index in a reaction mixture containing 5 μl of 5× Herculase II reaction buffer, 0.25 μl of 100 mM dNTP mix, 1.25 μl of dimethyl sulfoxide (DMSO), 0.5 μl of Herculase II Fusion DNA Polymerase, and 6 μl of water to a final volume of 25 μl. Each sample was dual indexed. The amplified libraries were purified with AMPure XP beads (Beckman Coulter Inc., Brea, CA, USA). The quality and quantity of amplified libraries were assessed using the Agilent 2100 Bioanalyzer (Agilent Technologies, Waldbronn, Germany) and DNA 1000 Assay. The final dual indexed libraries were pooled into one at equimolar concentrations. The library was subjected to multiplex sequencing on the MiSeq sequencer (Illumina).

### NGS sequence data analysis

Sequence data analysis followed a framework described previously [71]. Briefly, Fastq files were generated as row sequences by the Illumina MiSeq, with 600 cycles of paired-end read, and validated by evaluation of the distribution of quality scores. Validated fastq files from each viral genome were aligned with the Burrows-Wheeler Aligner tool (BWA v. 0.7.8-r455) [72] against a reference sequence and the resulting Alignment Map (SAM) format output was suitable for analyses using SAMTools [73]. Analysis of sequence quality, depth of coverage, short-read alignment, and variant identification were performed using SAMTools [73]. The indexed file was visualized using the Integrative Genomics Viewer (IGV) [74]. The BLV cell line FLK-BLV subclone pBLV913 (GenBank accession number: EF600696) [47], BLV strain Arg41 (GenBank accession number: FJ914764) [59], and BLV (GenBank accession number: AF033818) complete genomes were used as reference sequences. Full genome consensus sequences of each individual BLV strain were saved for further analyses, including alignment using MAFFT v7.123b (http://mafft.cbrc.jp/alignment/software/) and deduction of protein sequences by in silico translation of nucleotide to amino acid sequences using MEGA 5.1 [68].

BLV genome sequences (8728 bp) were aligned using MAFFT software, and the nucleotide and amino acid substitution per site was calculated using the Jukes and Cantor [75], and *p*-distance models [68], respectively. A ML tree for full BLV genome sequences was constructed using MEGA 6.06 software [76], and 1000 replications were used to calculate bootstrap values.

## Additional files

10.1186/s12977-016-0239-z Strategy and primers used for the amplification of full-length BLV genomes.
10.1186/s12977-016-0239-z Average sequencing depth for 25 aligned BAM files generated from 25 samples sequenced by MiSeq sequencer.
