# Supplementary material for: A new genotype of bovine leukemia virus in South America identified by NGS-based whole genome sequencing and molecular evolutionary genetic analysis
Source: Retrovirology. 2016 Jan 12;13:4. doi: 10.1186/s12977-016-0239-z (PMC4709907; doi:10.1186/s12977-016-0239-z)
Supplement: Supplementary file 1 — 10.1186/s12977-016-0239-z Strategy and primers used for the amplification of full-length BLV genomes. [file 12977_2016_239_MOESM1_ESM.docx]

Additional File 1: Figure S1. Strategy and primers used for the amplification of full-length BLV genomes

| Amplified position | | Primers (Binding position) | Sequence | Length of amplicon |
| --- | --- | --- | --- | --- |
| 1 | | LRT-F (2-23nt; 8201-8222nt) | 5`-GTATGAAAGATCATGCCGACCT-3` | 806 bp |
|  |  | 807-R (781-807nt) | 5`-CCAGAAGTGAAAGTCCATGGTT-3` |  |
| 2 | 1st PCR (External) | 94F (94-113nt) | 5`-GGCTAGAATCCCCGTACCTC-3` | 5760 bp |
|  |  | 5853R (5834-5853nt) | 5`-TGATCTTGCTCCAGAACGTG-3` |  |
|  | *2nd PCR*  (External) | 741F (741-765nt) | 5`-CCGATTTAAAGAATTACATCCATTG -3` | 3877 bp |
|  |  | 4617R (4601-4617nt) | 5`-CCCATCTGGTCTTTAGAATTGG-3` |  |
| 3 | | 4380F (4380-4403nt) | 5`-AACTACACCTCCAAAACCTTTGTC-3` | 3309 bp |
|  |  | 4380-G2-F (4380-4403nt)* | 5`- AACTACACCTCTAGAACCTTTGCC-3` |  |
|  |  | 7688R (7665-7688nt) | 5`-GAGGACAGGATGCGTTACTAAGTT-3` |  |
| 4 | | 7397F (7397-7416nt) | 5`-CGAGACCCACCGTATCAACT-3` | 1280 bp |
|  |  | LRT-R (455-477nt; 8654-8676 nt) | 5`- GCGAGAAACAGAAAGTAAGACAGG-3` |  |

A scheme on the top shows the strategy for amplifying the BLV genome in four PCRs (1-4) using appropriate primers shown in the table. The full length of BLV complete genome were shown as numbers in the beginning and ending of the scheme. The binding position of the primers were also shown in the table.. Binding position refers to Bovine leukemia virus cell-line FLK-BLV sub-clone pBLV913 complete genome (Accession number: EF600696 ).

* indicates the forward primer used specifically for the amplification of the fragment from 4380 nt to 7688 nt of South American BLV genotype-2 strains.

Numbers with nt indicates the PCR amplification region shown as the starting and ending nucleotide position.

Square dot box 5 and 6 indicate the sequences of the region was obtained from NGS.
