# Supplementary material for: A new genotype of bovine leukemia virus in South America identified by NGS-based whole genome sequencing and molecular evolutionary genetic analysis
Source: Retrovirology. 2016 Jan 12;13:4. doi: 10.1186/s12977-016-0239-z (PMC4709907; doi:10.1186/s12977-016-0239-z)
Supplement: Supplementary file 2 — 10.1186/s12977-016-0239-z Average sequencing depth for 25 aligned BAM files generated from 25 samples sequenced by MiSeq sequencer. [file 12977_2016_239_MOESM2_ESM.docx]

Additional File 2: Table S1. Average sequencing depth for 25 aligned BAM files generated from 25 samples sequenced by MiSeq sequencer.

| BAM file | Sample name | Average of depth |
| --- | --- | --- |
| Portachuelo108-new  Portachello-14b  Portachello-2b  Portachello-20b  Portachello-25b  Portachello-28b  Portachello-46b  Portachello-57b  Portachello-71b  Portachello-84b  Portachello-87b  Portachello-93b  Monetro-41b  Monetro-1b  Monetro-22b  Monetro-28b  Monetro-17b  Lima40G2  Paraguay17G1  Asuncion1-1G1  Asuncion5-1G1  Paraguay7G1  Paraguay62-2G6  Paraguay89-1G6  Paraguay91-1G6 | Portachuelo 108  Portachuelo 14  Portachuelo 2  Portachuelo 20  Portachuelo 25  Portachuelo 28  Portachuelo 46  Portachuelo 57  Portachuelo 71  Portachuelo 84  Portachuelo 87  Portachuelo 93  Montero 254  Montero 214  Montero 235  Montero 241  Montero 230  Lima40  Asuncion 43  Asuncion 1  Asuncion 5  Asuncion 41  Asuncion 62  Asuncion 89  Asuncion 91 | 317  953  1270  1190  1075  1190  858  1321  1094  952  1558  1349  895  1248  669  818  1013  396  595  718  425  495  364  1492  143 |
